# The Immune Response Is Involved in Atherosclerotic Plaque Calcification: Could the RANKL/RANK/OPG System Be a Marker of Plaque Instability?

**DOI:** 10.1155/2007/75805

**Published:** 2007-11-22

**Authors:** Fabrizio Montecucco, Sabine Steffens, François Mach

**Affiliations:** Division of Cardiology, Foundation for Medical Researches, University Hospital of Geneva, 1211 Geneva, Switzerland

## Abstract

Atherogenesis is characterized by an intense inflammatory process, involving immune and vascular cells. These cells play a crucial role in all phases of atherosclerotic plaque formation and complication through cytokine, protease, and prothrombotic factor secretion. The accumulation of inflammatory cells and thus high amounts of soluble mediators are responsible for the evolution of some plaques to instable phenotype which may lead to rupture. One condition strongly associated with plaque rupture is calcification, a physiopathological process orchestrated by several soluble factors, including the receptor activator of nuclear factor (NF)κB ligand (RANKL)/receptor activator of nuclear factor (NF)κB (RANK)/osteoprotegerin (OPG) system. Although some studies showed some interesting correlations with acute ischemic events, at present, more evidences are needed to evaluate the predictive and diagnostic value of serum sRANKL and OPG levels for clinical use. The major limitation is probably the poor specificity of these factors for cardiovascular disease. The identification of tissue-specific isoforms could increase the importance of sRANKL and OPG in predicting calcified plaque rupture and the dramatic ischemic consequences in the brain and the heart.

## 1. INTRODUCTION

During the recent years, atherogenesis has been well known as an intense inflammatory systemic process, involving immune and vascular cells [1]. The anatomic structure of atherosclerotic plaques is well known. The atherosclerotic plaque is localized in the arterial intima and contains immune cells (T cells, B cells, NK cells, monocyte/ macrophages, mast cells, dendritic cells), foam cells, vascular endothelial cells, and smooth muscle cells [2–5], that are around a core of lipids, extracellular matrix and lipid-rich debris from dead cells [1]. All these cells play a crucial role in all phases of atherosclerotic plaque formation and complication through T_H_1-type cytokine, protease, and prothrombotic factor secretion [2]. On the other hand, despite these proatherosclerotic activities, these cells are also capable of attenuating the maturation of atherosclerotic plaques, through the production of anti-inflammatory cytokines, such as TGF-β and IL-10 [6, 7]. In particular, a subpopulation of T cells, called CD4^+^CD25^+^ regulatory T cells (T_reg_), was recently shown to reduce atherosclerosis in ${\text{ApoE}}^{-/-}$ mice [8, 9]. A fibrous cap of smooth muscle cells and collagen fibres surrounds the complex pro/anti-inflammatory tissue (called lipid core); and an endothelial cell layer divides the plaque from the blood stream [1]. The plasticity of these cells and the great variety of soluble mediators are responsible for the evolution of some plaques to instability, with high risk of fibrous cap disruption and the subsequent acute ischemic and thrombotic events, such as artery occlusion or arterial embolism. One condition strongly associated to plaque rupture is calcification [10–13]. In fact, the degree of calcification promotes the number of interfaces between rigid and distensible portions of the plaque until the point of rupture. This suggests that dystrophic calcification at the thin fibrous cap [14], rather than the histological appearance of fully formed bone with trabeculations of the plaque [15], is related to the increased risk of plaque rupture with the consequent dramatic ischemic events [16]. Monocytes, dendritic cells, and smooth muscle cells are crucial for calcium deposition in the lesion, because of their retained capability to differentiate into osteoblast-like cells and osteoclast-like cells [17–22]. These cells, controlled by cytokines and other soluble factors, are the key players of the calcification process.

## 2. CURRENT STRATEGIES TO REDUCE PLAQUE CALCIFICATION

During the last decades, some unstandardized treatments have been proposed to reduce the maturation of the plaque towards calcification. Given the involvement of immune cells, an immunosuppressing pharmacological approach was attempted with some significant results. For instance, in preclinical studies, cyclosporin was found capable of reducing intimal cell proliferation after arterial injury [23]. In addition, clinical studies suggested that sirolimus and statins reduce atherosclerotic complications [24, 25]. Employing a different strategy, researchers focused their attention on molecules capable of reducing atherosclerotic risk factors. Beta blockers and estrogens were found capable of reducing the development of calcification in coronary arteries [26, 27]. No clear evidences for antiatherosclerotic activities are actually attributed to the ligands for peroxisome-proliferator-activated receptors (PPARs), the nonsteroidal anti-inflammatory drugs (NSAIDs), and bisphosphonates, because there were controversial effects between in vitro and in vivo experiences [28–33]. All these pharmacological molecules were focused on modulating the innate and adaptive immunity to reduce the inflammatory processes, and thus preventing plaque calcification. On the other hand, Price and coworkers also proposed a new therapeutic approach, focused on arterial calcification physiopathology. They performed a treatment with 1 mg/day osteoprotegerin (OPG) for inhibiting artery calcification induced by Warfarin and by vitamin D in mice and they obtained a dramatic reduction of calcification of arteries [34]. Although the real role of OPG as a cardiovascular risk factor is not well clarified and further studies are needed, the use of OPG could be a very promising therapeutic strategy based on arterial physiopathology. Another approach independent of CD4^+^ T cell activation was recently performed. For instance, ${\text{Ldlr}}^{-/-}$ mice vaccinated with malondialdehyde-modified LDL; and HSP60 demonstrated some encouraging preliminary results [35, 36]. Intriguingly, these interventions strongly support the importance of humoral immunity in atherosclerotic processes. The modulation of both innate and adaptive immunity may be a useful strategy to reduce the development of atherosclerotic plaque calcification. The development of new therapeutic approaches is needed because when established, arterial calcifications are irreversible [37] and, despite controversies, only the surgical treatment remains [38]. For all these reasons, new therapies capable of reducing established and developing calcification of the plaque need to be developed to reduce acute ischemic cardiovascular events, independently of traditional risk factors [39–43]. The present review is focused on identifying molecular mechanisms and serological markers to better characterize the cardiovascular risk and possible targets for future therapies against arterial calcification and the consequent plaque rupture.

## 3. MOLECULAR MECHANISMS OF ARTERIAL CALCIFICATION

Although previously considered as a passive precipitation, recent work suggests that calcium mineral deposition in atherosclerotic plaques is the result of intra-arterial processes of osteogenesis [10]. Despite considerable confusion, in 2004 Doherty et al. had identified two different types of arterial calcification, localized in the media or the intima, respectively [44]. Medial and intimal calcifications are different entities that are not necessarily separated from each other. In fact, medial calcification occurs independently of atherosclerosis [45], and is observed with high frequency in Monckeberg's sclerosis [46], hypervitaminosis D [47], end-stage renal failure disease (ESRD) [48, 49], and diabetes mellitus [50, 51]. Although the precise mechanism of medial calcification is not clear, at least for ESRD, an association between arterial calcification and increased serum phosphorus and increased ion product [${\text{Ca}}^{2+}\times {{\text{PO}}_{4}}^{-3}$] was shown [52]. In diabetes mellitus, different hypotheses for medial calcification formation were formulated. For instance, Edmonds suggested a possible involvement of stiffening of arterial tone and endothelial dysfunction [53]. However, much remains to be investigated about medial arterial calcification, such as a possible association with the cardiovascular risk [54, 55].

On the other hand, intimal calcification was observed almost exclusively in atherosclerotic plaques [10], and it occurs in two distinct patterns (punctate or diffuse), with still unclear implications [44]. So far, several molecular mechanisms of plaque calcification have been identified, with many similarities to physiopathological processes of bone formation [56] and resorption [57]. Intimal arterial calcification might be secondary to an imbalance between these two opposing processes, with the inhibition of osteoclast-like (OCL) cell mineral resorption and the increase of osteoblast-like (OBL) cell mineral deposition [58]. In the following, we will discuss three different models of plaque calcification that have been proposed by Doherty and colleagues, with complementary molecular mechanisms, which might be involved [59].

### 3.1. The “active model” of arterial calcification

In 1993, Bostrom et al. showed the presence of pluripotent arterial cells, called calcifying vascular cells (CVCs), which are immunologically distinct from the other arterial cells [60]. These cells colocalized in atherosclerotic plaques with bone-related proteins and transcriptional factors, such as BMP-2 and Cbfa1 [60, 61]. Furthermore, they were found capable of forming in vitro mineralized structures [62, 63]. These data were confirmed by other groups, which extended the active model of bone matrix formation also to other arterial cell types, such as smooth muscle cells [64–66]. The name “active model” is derived from the bone formation activity of these cells, also called OBL cells. The validity of the present model was also confirmed by in vivo experiences showing that both human and animal artery mineralization processes are very similar to that observed in bone [67–69].

### 3.2. The “passive physicochemical model” of arterial calcification

This model was proposed by Gijsbers et al. [70] and Schinke and Karsenty [71] and is based on the concept that calcium and phosphate ions are in a metastable state when they are near the point of precipitation in solid phase within biological fluids. Vermeer showed that several proteins, which chelate calcium cations, inhibited mineral salt deposition in arteries. These proteins (mainly homeostatic clotting factors and osteocalcin) were found to contain glutamine residues carboxylated at the γ-position gamma-carboxyglutamic acid (Gla) residues, and thus were called Gla proteins [72]. In accordance with this model, atherosclerotic plaque calcification is due to a deficient chemical γ-carboxylation of Gla proteins. This “passive” model is mainly supported by two several independent findings. First, the enzyme γ-carboxylase was found less active in atherosclerotic rather than in normal arteries in both humans and animals [13, 73]. This may be due to a deficiency of the two cofactors (two isoforms of vitamin K, named phylloquinone and menaquinone), needed for the chemical reaction [74]. Secondly, mice deficient for matrix gamma-carboxyglutamic acid (Gla) protein (MGP) showed a massive arterial calcification [75].

On the other hand, other studies raised several doubts on the real relevance of the passive model of arterial calcification. For instance, in Keutel syndrome, a human disease characterized by a nonfunctional MGP gene, patients do not develop a massive arterial calcification [73]. In addition, MGP knockout mice and rats develop medial rather than intimal calcification, which characterizes atherosclerosis [75]. Therefore, a combined role of MGP deficiency with other factors has been suggested. Moreover, the cysteine protease inhibitor AHSG [76], apoptotic bodies [77], and lipids [78] were found to be important modulatory factors of atherosclerotic intimal calcification. To summarize, current evidence suggests that the “passive” model of calcification appears to be relevant mainly in medial calcification, a histological entity not clearly related to atherosclerosis.

### 3.3. The arterial OCL model

We have previously described that bone remodelling results from the balance between formation (osteoblasts) and degradation (osteoclasts). While the “active model” highlights the importance of OBL cells, the “arterial OCL model” proposes that arterial calcification is due to a lack of activity of OCL cells. Several molecular factors influence OCL survival, differentiation, and function. Macrophage-colony stimulating factor (M-CSF), a cytokine, and growth factor for mononuclear phagocytic cells (MPCs) is crucial in survival and differentiation of osteoclast progenitors [57, 79]. This role is strongly supported by independent evidences, showing that the lack of M-CSF alone was sufficient to reduce the number of osteoclasts and induce osteopetrosis [80–82]. In addition, despite a significant reduction of atherosclerotic lesion formation, mice deficient for both M-CSF and apoliprotein (apo) E-developed plaque calcification [83]. These data highlight the dual role of M-CSF in atherosclerosis: the promotion of atherogenesis (plaque formation) and the inhibition of plaque calcification (plaque complication).

**Figure 1:**
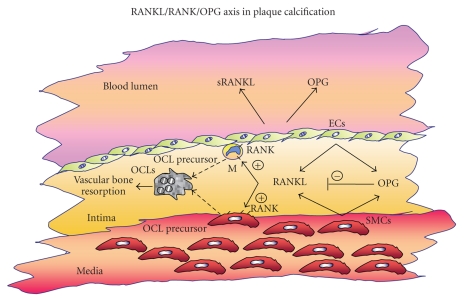
**Schematic diagram of potential OCL differentiation in the plaque.** Soluble RANKL (sRANKL) and OPG are secreted in the atherosclerotic plaque and in the blood stream mainly by smooth muscle cells (SMCs) and endothelial cells (ECs). sRANKL promotes OCL precursor (mainly monocytes/macrophages (M), dendritic cells, and SMCs) differentiation into OCL cells (as indicated by dotted arrows). OPG neutralizes the action of RANKL. The balance between these two soluble molecules regulates the bone resorption in calcified plaques, which is correlated to plaque rupture.

On the other hand, the receptor activator of nuclear factor (NF)κB ligand (RANKL), which is also called tumor necrosis factor- [TNF-] related activation-induced cytokine (TRANCE) or osteoprotegerin ligand (OPGL) [84], is also necessary and sufficient for the generation and function of OCL cells in the plaque (Figure 1). RANKL, which is expressed in unstable atherosclerotic plaques [85–87], is capable of modulating different cell-type activities (mainly monocyte-derived osteoclast precursors, T cells, B cells, and dendritic cells) [88, 89] through its transmembrane receptor RANK. After the binding with RANK, several intracellular signal transduction pathways are activated, with crucial role for mitogen-activated protein kinases (MAPKs) and (NF)κB [90, 91]. Taking into the account these premises, RANKL appears as an anticalcifying molecule, and probably capable of reducing the plaque vulnerability. Some of these findings were not confirmed by Sandberg and colleagues [87], showing surprisingly that RANKL induces plaque instability in humans by inducing MCP-1 and matrix metalloproteinase (MMP) production [87]. Thus, the exact role of RANKL in plaque dystrophic calcification remains unclear. In fact, in absence of the RANKL neutralizing agent OPG, the decoy receptor of RANKL, mice not only developed osteoporosis (bone loss), but also arterial calcification [92]. There are at least two explanations suggesting a different role of RANKL between human and mice. First, although expressed in human arteries, RANKL, and RANK are not expressed in normal mouse arteries, but only in calcified plaque [93]. This suggests that the calcification process itself might upregulate RANK and RANKL expression and signalling. In this case, RANKL-induced OCL anticalcification activity is secondary to the establishment of a consolidated calcification, without involvement in plaque formation and maturation, at least in mice. Second, RANKL signalling can also promote mineral deposition in mouse plaques. This interesting hypothesis is sustained by Lin et al., showing osteoblast proliferation in murine calvarial organ culture [94]. On the contrary, evidences showed RANKL and OPG presence and activity in early and advanced atherosclerotic lesions in humans [95, 96]. The soluble form of RANKL and serum OPG detected in human blood stream (mainly released from endothelial cells) are both under investigation as possible clinical biomarkers of several bone-related diseases, including atherosclerosis [97, 98]. Therefore, even though the full mechanism of bone resorption is still not clarified, the “OCL model” has to be considered as directly involved in intimal plaque calcification through the active inhibition of calcification and the degradation of existing mineral deposits.

## 4. MOLECULAR FACTORS INVOLVED IN ATHEROSCLEROTIC ARTERIAL CALCIFICATION

Although several studies on arterial calcification have been performed, the molecular mechanisms influencing bone metabolism are still unclear. Bone remodelling is a process common to various diseases, often coexisting. The real difficulty is to define a biomarker specific only for the cardiovascular risk of plaque rupture and not influenced by osteoporosis, renal failure, or other bone-related diseases. For these reasons, several parameters altered or involved in bone metabolism have been studied. Investigators started with parathyroid hormone (PTH) and vitamin D, which are the principal factors for bone homeostasis. Discordant results were obtained [99–101] and actually no clear correlation between PTH, vitamin D, and vascular calcification were observed. On the other hand, given the low incidence of coronary heart disease (CHD) in premenopausal women [102], estrogens were investigated. Substantial evidence showed that estrogens have an antiatherogenic effect, mainly through lipid-lowering [103] and endothelial nitric oxide synthase (eNOS) activation [104]. A direct casual relationship with estrogens and arterial calcification has been shown recently [27], even though further evidences are needed. Lipid metabolism and leptin were studied as possible markers of plaque calcification, but no direct correlations were identified [105]. Other markers were analysed by Doherty et al., but they require further studies to better elucidate their potential importance [44]. Among these markers, the RANKL/RANK/OPG system appears as the most promising for an application in the near future.

## 5. COULD THE RANKL/RANK/OPG SYSTEM BE CONSIDERED AS A SEROLOGICAL MARKER FOR PLAQUE RUPTURE IN THE FUTURE?

As previously described, there is strong evidence for an implication of the RANKL/RANK/OPG network in vascular calcification. However, atherosclerotic arterial calcification shares the activation of this system with other pathologies, such as rheumatoid arthritis, osteoporosis, cancer metastasis [106, 107], and other vascular diseases, such as diabetic macroangiopathy, aortic aneurism, and heart failure [108]. Several studies indicate that the RANKL/RANK/OPG axis is not specific for plaque calcification and destabilization. Nevertheless, OPG and sRANKL serum levels have been proposed as biomarkers of vascular risk and prognosis. The serum levels of OPG were measured in patients with cerebrovascular disease, stable angina, and coronary artery disease (CAD), and showed interesting correlations. In particular, OPG levels were independently associated with cardiovascular mortality, but not bone mineral density in patients suffering from cerebrovascular diseases [109]. Furthermore, OPG is correlated with significant coronary artery narrowing [110]. Interestingly, osteoprotegerin gene polymorphisms were shown in coronary artery disease in Caucasian men [111]. Finally, serum OPG levels were associated to the severity of CAD [112]. However, although further clinical studies are needed to confirm that serum OPG levels might help to evaluate the prognosis of vascular disease. Serum levels of free- (not complexed to OPG) soluble RANKL (sRANKL) were also found altered in CAD patients. In particular, they were significantly lower in patients with CAD, without reporting a correlation to the severity of the disease [113]. These findings were also confirmed by Jono et al. [112] and Sandberg et al. [87], demonstrating the increase of levels of RANKL expression in T cells during acute coronary syndrome [87]. Although OPG and free-soluble RANKL might be considered as a promising marker of cardiovascular risk, their application might be limited by poor tissue specificity. For this reason, the identification of tissue-specific isoforms of OPG and RANKL could contribute to highly increase future diagnostic and prognostic significances.

## 6. CONCLUSIONS

The present review shows that plaque calcification represents a crucial step for plaque destabilization and rupture. Some serological markers are needed to be validated for better defining cardiovascular risk and prognosis of acute ischemic complications, secondary to plaque rupture. Although not selective only for arterial calcification, the RANKL/RANK/OPG axis could be a promising risk marker and target for future therapies. In this context, experimental data have provided the first evidence for the therapeutic use of OPG as possible pharmacologic agent for reducing arterial calcification [34]. On the contrary, human data suggested the direct relationship between increased OPG serum levels and plaque destabilization. This may imply that elevated OPG levels could be compensatory rather than causational in atherosclerotic calcification. Therefore, further clinical investigations with large number of patients are required to better clarify the role of serum sRANKL and OPG in plaque physiopathology.
